# A versatile ceramic capillary membrane reactor system for continuous enzyme‐catalyzed hydrolysis

**DOI:** 10.1002/elsc.202100027

**Published:** 2021-07-09

**Authors:** Lorn Messner, Marieke H. Antink, Tongwei Guo, Michael Maas, Sascha Beutel

**Affiliations:** ^1^ Institute for Technical Chemistry Leibniz University Hannover Hannover Germany; ^2^ Advanced Ceramics University of Bremen Bremen Germany; ^3^ MAPEX Center for Materials and Processes University of Bremen Bremen Germany

**Keywords:** antibody fragments, bioactive peptides, food proteins, immobilized enzymes, proteolysis

## Abstract

As an alternative to classical batch processes, enzyme‐catalyzed hydrolysis can also be carried out continuously. To facilitate this, a continuous ceramic capillary membrane reactor system (CCCMRS) was developed which can be operated with various proteolytic enzymes immobilized on the porous ceramic capillary membranes. This system has several advantages over common batch processes regarding stability, reproducibility and controllability and can easily be adapted to optimal reaction conditions and individual preferences. Two exemplary applications utilizing the CCCMRS were carried out and investigated in long‐term stability studies. In the first application the continuous enzymatic cleavage of human IgG into the antibody fragments Fab and Fc by immobilized papain was performed. A total volume of 22 mL of 1 mg mL^‐1^ IgG‐solution was enzymatically cleaved over a period of 33.3 h. The antibody cleavage products could be detected in an SEC‐HPLC over the whole process time thus indicating long‐term stability of the continuous hydrolysis process. The second application investigated the continuous digestion of pea and almond protein isolates by immobilized Alcalase resulting in the generation of a large variety of different peptides. This peptide fingerprint remains constant over a long period of time enabling fractionation and thus making the peptides accessible for further bioactivity studies in sufficient quantities. The constant peptide fingerprint could be shown in the RP‐HPLC analysis for all 30 samples with a total volume of 29.7 mL collected over a period of 45 h.

**Abbreviations**
AUC: area under the curve
BANA: N‐α‐benzoyl‐DL‐arginine β‐naphthylamide hydrochloride
BAP: bioactive peptide
Boc‐Ala‐ONP: Boc‐l‐alanine‐4‐nitrophenyl ester
CCCMRS: continuous ceramic capillary membrane reactor system
CNT: carbon nanotubes
DMSO: dimethyl sulfoxide
FPLC: fast protein liquid chromatography
GO: graphene oxide
IgG: immunglobulin G
MOF: metal organic framework
PBR: packed‐bed reactor
PES: polyethersulfon
RP: reversed phase
SEC: size exclusion chromatography
TFA: trifluoroacetic acid

## 1 INTRODUCTION

Enzyme‐catalyzed hydrolytic processes are key processes in many fields of modern bioprocessing, e.g. for the cleavage of His‐tags or other fusion proteins, the maturation of precursor proteins, the generation of antibody fragments and the production of peptides. Usually, these processes are carried out batch‐wise, which is operator‐intensive and often leads to batch‐to‐batch variations [1]. Carrying out such enzyme catalyzed hydrolysis processes in continuous reactor systems with immobilized enzymes would have several advantages compared to that of batch processes.

The number of continuous flow reactor systems described in the literature has significantly increased within the last few years. The possibility of easy automatization, increasing global awareness for sustainability and stricter chemical safety regulations raised the interest in continuous flow reactor systems as regeneration is possible and the handling of hazardous substances is simplified. Continuous flow reactor systems like the packed‐bed reactor (PBR) can be applied in several scientific fields like chemistry, biotechnology, biomedicine and photocatalysis in laboratory scale as well as in industrial production processes [2]. Recent studies on enzyme‐immobilized biocatalytic membranes focus on novel nanomaterials suitable for the immobilization of enzymes like proteases, peroxidases, carbonic anhydrases, lipases, β‐galactosidases and pectinases which are commonly used in biocatalytic processes. Materials like carbon nanotubes (CNTs), graphene oxides (GO) and metal organic frameworks (MOFs) enable stable and high‐density enzyme‐immobilization, high activity and easy regeneration [3]. Lin et al. used GO‐based nanomaterials for immobilizing formate dehydrogenase observing high stability and recovery yield ensuring a high grade of reusability [4]. Ji et al. immobilized laccase on a polymeric membrane coating of CNTs using electrostatic interaction. The partially inactivated laccase could be washed off the CNTs coating layer with surfactant solution and then be reloaded onto the coating maintaining a large part of the initial enzyme activity [5]. Other studies examined different biocatalytic membrane reactor designs. Online tryptic protein digestion of BSA, myoglobin, and cytochrome c has been performed in a silica‐based immobilized enzyme reactor (IMER) in the presence of an organic mobile phase. This approach combines the principle of a packed bed reactor with an RP‐chromatography column. Despite an excellent reproduction rate, the compatibility of some proteins with an organic solvent led to difficulties [6]. A different strategy was pursued by stacking numerous cellulose membranes immobilized with proteolytic enzymes. This enzyme‐membrane reactor system (EMR) was examined regarding long‐term stability by performing a peptic digest of casein. Although long‐term stability could be observed for a duration of four days, difficulties still occurred regarding protein aggregation in the reactor system as well as foam generation of the protein solution [7].

Nevertheless, employing these reactor systems for proteolytic applications simplifies further purification steps since the immobilized proteases do not remain in the hydrolysate. The continuously‐generated hydrolysis product shows a constant and stable peptide fingerprint which enables the specific production and subsequent fractionation of defined peptides, antibody fragments or maturated enzymes from inactive precursors. By simply adjusting parameters such as flow rate, temperature and pH‐values, a specific process can be varied to find optimal reaction conditions and be adapted to individual preferences.

**PRACTICAL APPLICATION**

Continuous processing of enzyme‐catalyzed hydrolysis can be achieved by application of a ceramic capillary membrane reactor system (CCCMRS) decorated with the respective proteolytic enzymes. Two exemplary applications are highlighted in this article: The first application examines the enzymatic cleavage of antibodies into fragments which is a procedure frequently used in the pharmaceutical industry for the production of antibody fragment‐based therapeutics. Using a continuous hydrolysis process with immobilized proteolytic enzymes can avoid batch‐to‐batch variability in critical pharmaceutical production steps and can in addition facilitate the following purification steps. The second application focuses on the enzyme‐catalyzed hydrolysis of food proteins from various sources such as pea, almond and lupine. This is a promising method applied in drug discovery and research. It serves to generate bioactive peptides encrypted within several native proteins contained in the aforementioned sources. The continuous production of potentially bioactive peptides with immobilized serine‐proteases offers a combination of excellent controllability, high purity and reproducibility.

Our previous studies on continuous reactor systems utilizing immobilized proteolytic ceramic capillary membranes have already proven their suitability for continuous proteolytic applications [8, 9]. The continuous ceramic capillary membrane reactor system (CCCMRS) presented in this study was further optimized concerning long‐term stability and production yield. Two exemplary applications prove the suitability of the system as a platform technology for enzyme‐catalyzed hydrolysis processes.

The immobilization of proteolytic enzymes to the highly porous ceramic capillary membranes was performed using carbodiimide cross‐linker chemistry. This results in a covalent bond between the primary amine of the APTES‐linker molecule bound to the surface of the ceramic capillary membrane and the carboxyl‐functionalization of the proteolytic enzyme [10]. Covalent binding of the proteolytic enzymes to the APTES‐linker molecule was proven to be the most stable bond with the highest enzyme activity in previous studies [8]. Long‐term stability studies were performed utilizing the CCCMRS for two exemplary applications which are described in detail in the following.

**FIGURE 1:**
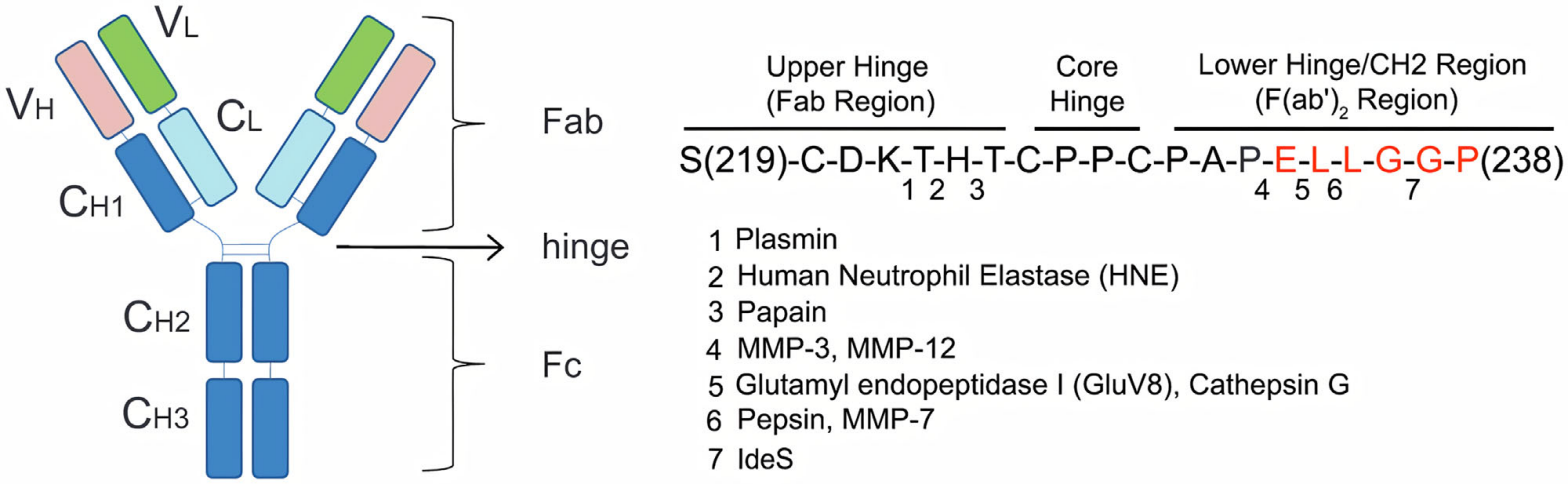
Human IgG cleavage regions of different proteases [11]. Papain selectively cleaves between histidine and threonine in the upper hinge region of human IgG antibody

In the first application, the hydrolytic generation of Fab‐fragments from Immunoglobulin G by using papain‐immobilized capillary membranes was examined. Papain is a thiol‐type endoprotease with a specificity for the cleavage between histidine and threonine in the upper‐hinge region of IgG. This results in the formation of IgG‐Fab and Fc‐fragments (Figure 1) [11]. The usage of antibody Fab‐fragments has several advantages over the usage of the intact antibody. Since many cells have Fc‐region‐binding receptors, nonspecific binding can be reduced by the removal of the Fc‐region of the antibody. Moreover, the smaller IgG‐Fab‐fragments allow a more efficient penetration of biological membranes. This leads to improved staining abilities, e.g. in immunohistochemistry. The usage of a less complex system also simplifies further structural studies such as NMR or X‐ray crystallography. IgG‐Fab‐fragments also have a lower immunogenicity than the intact antibody, thus playing an essential role in in vivo experiments. Antibody Fab‐fragments also play an important role as therapeutics (Table 1) [12].

**TABLE 1:** Therapeutic monoclonal antibody fragments approved in the US [12]

Generic names	Description	Indication	Date of approval (US)	Sponsor
Abciximab	Anti‐GPIIb/IIIa chimeric Fab	Clot prevention in angioplasty	22.12.94	Centocor
Ranibizumab	Anti‐VEGF‐A humanized Fab	Macular degeneration	30.06.06	Genentech
Certolizumab pegol	Anti‐TNFalpha pegylated humanized Fab	Moderate to severe Crohn disease	22.04.08	UCB

In the second application, the CCCMRS was utilized for the continuous hydrolysis of different food and plant protein isolates of pea and almond origin. The ceramic capillary membranes were therefore immobilized with Alcalase, a bacterial, alkaline serine‐protease with a broad specificity occurring naturally in the extracellular matrix of *Bacillus licheniformis*. This protease shows excellent activity at physiological pH‐values and generates a homogenous distribution of various peptide lengths. As the protease belongs to the group of endopeptidases, the hydrolysis with Alcalase does not result in the formation of single amino acids [13]. The peptides within the hydrolysate can thereafter be fractionated using preparative chromatography and then utilized for further bioactivity studies in drug research and discovery. This is a promising way of finding new BAPs [14]. Besides using enzyme‐catalyzed hydrolysis, BAPs can also be generated with the help of biological libraries and chemical synthesis [15, 16]. Antioxidative effects as well as α‐glucosidase and pancreatic lipase inhibition have already been shown for Alcalase digests of almond protein isolate [17]. Pepsin was shown to generate stable almond protein isolate digests with excellent physical properties [18]. A tryptic digest of pea protein isolate was tested and found to have an anti‐adhesive activity to *Helicobacter pylori* [19]. Several research groups have already discovered various BAPs originating from food or plant protein (Table 2) [20].

**TABLE 2:** Bioactive peptides with their origin and sequence [20]

Bioactivity	Origin	Sequence	Reference
Antioxidative	Soybean	LVNPHDHQNLK	Capriotti et al. (2015) [39]
	Egg	DEDTQAMP	Nimalaratne et al. (2015) [40]
Antihypertensive	Algae	AKYSY	Harnedy & Fitzgerald (2011) [41]
	Milk	LHLPLP	Quirós et al. (2006) [42]
Antimicrobial	Milk	FSDKKIAK	Capriotti et al. (2016) [43]
	Soybean	PGTAVFK	McClean et al. (2015) [44]
Anticarcinogenic	Soybean	GLLVDLL	Sanjukta & Ray (2016) [45]
	Chickpea	ARQSHFANAQP	Zhaohui Xue et al. (2015) [46]
Antidiabetic	Milk	MHQPPQPL	Zhang et al. (2015) [47]
	Algae	MAGVDHI	Harnedy et al. (2015) [48]

Due to the fact that autoproteolysis is a difficulty which commonly occurs at proteolytic enzymes in solution, immobilization to a solid carrier material can perceptibly enhance the stability [21]. Although enzyme immobilization usually leads to a slightly lower activity, it does make the process more durable and reliable [22]. An additional problem when working with protein solutions as a substrate for proteolytic enzymes also includes protein stability and aggregation, both of which can reduce the product quality and lead to decreased digestibility and therefore to a poor performance of the enzyme‐catalyzed hydrolysis process [23]. Long‐term studies have shown that the storage temperature and convective transport of the protein feed solution is a crucial factor concerning protein aggregation [24]. Combining continuous stirring together with cooling the protein feed solution significantly reduces protein aggregation and improves stability.

When working with a continuous flow system, the hydrolysate fingerprint essentially depends on the set flow rate as proved in a previous research project on CCCMRS [9]. Although working with the smallest possible flow rates can lead to the maximum degree of hydrolysis, this is not always desired in all applications so that it may be necessary to adjust the flow rate for each specific application. As all important parameters such as flow rate, temperature and pH can be adjusted precisely, the CCCMRS can be adapted to each specific enzyme‐catalyzed hydrolysis process.

The aim of this work was to perform different proteolytic applications on the CCCMRS to prove its suitability as a technology platform for enzyme‐catalyzed hydrolysis processes and examine the long‐term stability of the hydrolysate fingerprint. This was achieved by collecting subsequent hydrolysate samples over a defined timespan and analyzing and comparing the cleavage patterns from the RP‐HPLC chromatograms.

## 2 MATERIALS AND METHODS

The CCCMRS consists of a peristaltic pump, a ceramic capillary membrane in a reactor shell and an autosampler. The fully‐tempered protein solution feed has a cooling capacity of down to −20°C which can also be magnetically stirred at a speed of up to 1000 rpm. The maximum reactor capacity is 100 mL. The peristaltic pump (0.38 mm inner Ø tubing) can be set to flow rates from 11 to 5000 μL min^−1^. The ceramic capillary membrane reactor is tempered in a column oven which can operate within a range from 10 to 100°C. The autosampler allows for the collection of a maximum of 180 samples within set time intervals and the cooling unit is able to freeze or cool the collected samples down to a minimum temperature of −20°C. The enzyme‐immobilized ceramic capillary membranes are closed with silicon at one end to ensure flow through the capillary membrane pores and to maximize the contact surface of the protein solution with the immobilized enzymes. After immobilization, the ceramic capillary membranes are allocated in the stainless steel reactor shell which is subsequently placed in the column oven at a set temperature.

### 2.1 Properties and preparation of the ceramic capillary membranes

**FIGURE 2:**
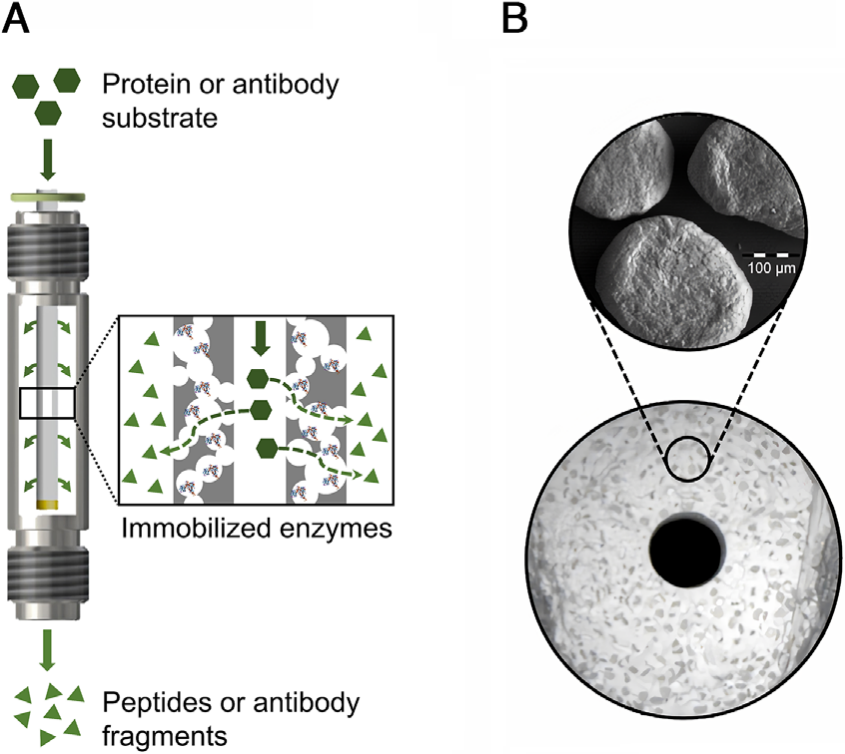
Reaction scheme of the immobilized ceramic capillary membrane reactor. Protein or antibody substrate flows through the capillary membrane pores and is cleaved due to surface contact with immobilized proteases resulting in the formation of peptides or antibody fragments (A) and cross‐section view of the ceramic capillary membrane with detailed visualization of the capillary membrane pores captured via polarized light‐microscope (B)

The tubular capillary membranes have been prepared according to our previous study [8]. In brief, the membranes are made of a ceramic and microcrystalline cellulose slurry which is extruded and sintered, leaving an average pore size of 90 nm. The sintered capillary membranes are then washed with deionized water and autoclaved at 121°C for 20 min before incubating them in 0.2 M APTES‐solution on a platform‐shaker at 65°C and 150 rpm for 24 h. After functionalization with the APTES linker molecule the capillary membranes are washed with deionized water to neutral pH and then dried at 70°C for 24 h. The total length of the final capillary membranes is 100 mm with a mass of 1.22 ± 0.069 g. The outer Ø is 5.43 ± 0.07 mm and the inner Ø is 1.18 ± 0.09 mm resulting in a total inner volume of 109.36 ± 17.32 μL. The specific surface area is 2.3 ± 0.35 m² g^−1^ and the open porosity is 63.68 ± 1.13% (Figure 2) [8]. The immobilization of the proteolytic enzymes on the ceramic capillary membranes was performed by way of NHS/EDC‐amine coupling as stated in the literature [25]. For the immobilization of one capillary membrane, 4.6 mg NHS and 12.4 mg EDC (Thermo Fisher Scientific, Waltham, USA) were dissolved each in 1 mL of immobilization buffer (0.1 M MES, 0.5 M NaCl, 0.1 M CaCl, pH 6, Sigma Aldrich, St. Louis, US) and then combined. After leaving the NHS and EDC to react for 10 min, 2 mL of protease solution (1 mg mL^−1^ in immobilization buffer) are added. To perform the coupling reaction, the capillary membrane is incubated at 4°C in the immobilization solution overnight for 16 h on the overhead shaker after quantifying the protein content of the solution via Pierce BCA Protein Assay Kit (Thermo Fisher Scientific, Waltham, USA). Immediately prior to use, the protein content of the immobilization solution was again quantified for the calculation of the immobilization yield. The capillary membrane is then rinsed thoroughly with the working buffer (20 mM POPSO, pH 7.8) to remove any excess immobilization solution. Before starting the hydrolysis process, the reactor system was purged using the working buffer at a flow rate of 2,000 μL min^−1^ for 30 min.

### 2.2 Determination of the enzyme activity of the native and immobilized enzyme

The enzyme activity of native and immobilized Alcalase was spectrometrically determined by observing the enzymatic conversion of Boc‐l‐alanine‐4‐nitrophenyl ester (Boc‐Ala‐ONP) to 4‐nitrophenol which can be detected at a wavelength of 405 nm. A stock solution of 15 mM Boc‐Ala‐ONP (Sigma Aldrich, St. Louis, US) in 80% ACN / 20% deionized H_2_O was prepared. After pulverizing an APTES‐functionalized ceramic capillary membrane the powder was immobilized with Alcalase according to the immobilization procedure described in Section 2.1. The immobilization yield was 75.24% which equals 1.5 mg of Alcalase. For the determination of the activity of immobilized Alcalase, the pulverized, immobilized ceramic capillary membrane was dispersed in 30 mL of working buffer by continuous stirring before 300 μL of the Boc‐Ala‐ONP solution were added to a final concentration of 150 μM. For the determination of the activity of native Alcalase, the equivalent amount of Alcalase immobilized to the ceramic capillary membrane was solved in 30 mL of working buffer before adding 300 μL of Boc‐Ala‐ONP stock solution to a final concentration of 150 μM. The solution was pumped through a Hellma Analytics low volume flow‐through cuvette with a flow rate of 1 mL min^−1^ after passing a 0.45 μm PES membrane filter to ensure that no particles can enter the cuvette. The spectrometric measurement was immediately started. The enzyme activity was calculated from the slope of the linear regression of the absorption over time.

The enzyme activity of native and immobilized papain was determined spectrometrically by observing the enzymatic conversion of N‐α‐Benzoyl‐DL‐arginine‐β‐naphthylamide‐hydrochloride (BANA) to 2‐Naphthylamine which can be detected at a wavelength of 540 nm. A stock solution of 15 mM BANA (Sigma Aldrich, St. Louis, US) in DMSO was prepared. The procedure for the determination of the activity of native and immobilized Alcalase was then applied for papain in the same way. The immobilization yield was 54.88% which equals 1.1 mg of papain. The calculated activities were 82.31 ± 1.41 U mL^−1^ (native Alcalase), 78.36 ± 1.18 U mL^−1^ (immobilized Alcalase) and 28.14 ± 0.8 U mL^−1^ (native papain), 23.88 ± 0.62 U mL^−1^ (immobilized papain).

### 2.3 Continuous hydrolysis of human immunoglobulin G

For the digestion process of human IgG, 25 mg of human IgG lyophilisate (Sigma Aldrich, St. Louis, US) was dissolved in 25 mL of working buffer, resulting in a solution with a final concentration of 1 mg mL^−1^. Cysteine‐HCl (AppliChem, Darmstadt, Germany) was added to a final concentration of 10 mM. After adjusting the pH to pH 7.8, the solution was injected into the protein solution feed flask which was constantly cooled to 4°C during the hydrolysis process. The ceramic capillary membranes were immobilized with papain (Sigma Aldrich, St. Louis, USA), whereas the immobilization yield was 49.75% resulting in an enzyme density of 0.53 μg cm^−2^ on the immobilized ceramic capillary membrane. The reactor was tempered to 37°C in the column oven. The peristaltic pump was set to a flow rate of 11 μL min^−1^. The sample collection time was set to 40 min resulting in a sample size of 440 μL per sample. In total 50 samples were collected over a 33.3 h period of time resulting in a total sample volume of 22 mL. The autosampler was cooled to −10°C ensuring instant freezing of the collected samples for the duration of the hydrolysis process in order to maintain the maximum stability up to the actual analysis in the SEC‐HPLC.

### 2.4 Continuous hydrolysis of pea and almond protein isolates

The digestion of the pea and almond protein solutions required the preparation of the appropriate protein isolate solutions using alkaline extraction. For this purpose, 5 g of pure pea and then almond protein (vegji organic protein isolate powder) were added respectively to 250 mL of working buffer with pH 12. The suspension was continuously rotated overnight at 4°C on a roller shaker. After centrifugation at 10,000 × *g* for 1 h, the supernatant liquid was removed and the pellet discarded. The pH of the collected supernatant liquid was adjusted to pH 7.8 with 3 M HCl before rotating again overnight at 4°C on a roller shaker. The centrifugation process was repeated whereupon the supernatant solution was finally filtered through a 0.22 μm PES‐membrane filter. The final protein concentration of the prepared solutions was determined by means of BCA‐Assay. The pea protein solution had a total protein concentration of 12.8 g L^−1^, the almond protein solution a total protein concentration of 7.9 g L^−1^. The ceramic capillary membranes were immobilized with Alcalase BG (Novozymes, Bagsværd, Denmark), whereas the immobilization yield was 68.67% resulting in an enzyme density of 0.76 μg cm^−2^ on the immobilized ceramic capillary membrane. The reactor was tempered to 40°C in the column oven. The peristaltic pump was set to a flow rate of 11 μL min^−1^. The sample collection time was set to 90 min resulting in a sample size of 990 μL per sample. A total of 30 samples were collected over a period of 45 h resulting in a total sample volume of 29.7 mL. The autosampler was cooled to 4°C to prevent protein aggregation in the collected hydrolysate.

### 2.5 Analysis in the HPLC system

Each collected sample of the IgG antibody hydrolysate was analyzed in a SEC‐HPLC using the YMC‐SEC mAB (3 μm) 300 × 4.6 mm column according to the YMC Biochromatography Columns Catalogue [26]. The eluent consisted of 0.1 M KH_2_PO_4_‐K_2_HPO_4_ containing 0.2 M NaCl. The flow rate was set to 0.165 mL min^−1^ and the column temperature to 25°C. A volume of 4 μL sample was injected into the column and detected at UV (280 nm). The isocratic elution was performed over a time of 45 min.

All samples collected from the hydrolysis of pea and almond protein solutions along with a peptide standard (HPLC peptide standard mixture, Sigma Aldrich, St. Louis, USA) were analyzed in a RP‐HPLC using the Phenomenex Aeris Peptide XB‐C18 (3.6 μm) 150 × 3.0 mm column. Eluent A consisted of deionized H_2_O/ACN/TFA (95:5:0.1) and eluent B of deionized H_2_O/ACN/TFA (20:80:0.1). The flow rate was set to 0.4 mL min^−1^ and the column was heated to 50°C. A 10 μL sample volume was injected into the column and detected at UV (214 nm). The gradient was set up as follows: 0–10% B (0–3 min), 10–50% B (3–55 min), 50–100% B (55–65 min), 0% B (65–75 min).

## 3 RESULTS AND DISCUSSION

**FIGURE 3:**
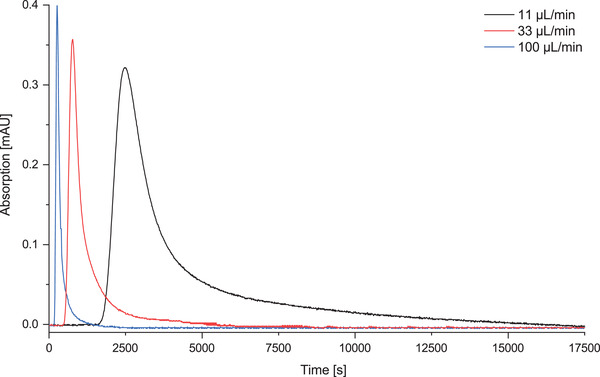
Spectrometric determination of the residence time distribution of 4‐nitrophenol as marker substance in the ceramic capillary membrane system at different flow rates

First, the CCCMRS was characterized taking into consideration the residence time distribution at different flow rates by injecting 100 μL of the tracer 4‐Nitrophenol via injection valve into the system. The absorption was spectrometrically measured at a wavelength of 405 nm until the absorption reached zero (Figure 3). At very low flow rates below 33 μL min^−1^ the residence time distribution showed a significant tailing whereas this effect decreased with higher flow rates. Reynolds numbers were calculated for the ceramic capillary membrane reactor with Re = 0.105 for a flow rate of 11 μL min^−1^, Re = 0.316 for 33 μL min^−1^ and Re = 0.959 for 100 μL min^−1^. Based on these calculations, the ceramic capillary membrane reactor shows a clear laminar flow pattern for low flow rates resulting in high momentum diffusion and low momentum convection. At lower flow rates the influence of diffusion processes, and thus the retention of substrate in the porous capillary membrane, increases. This explains the observation of pronounced tailing of the residence time distribution with lower flow rates. Working at a flow rate of 11 μL min^−1^, the protein solution behaves in the reactor system as shown in Figure 3.

### 3.1 Continuous hydrolysis of human immunoglobulin G

**FIGURE 4:**
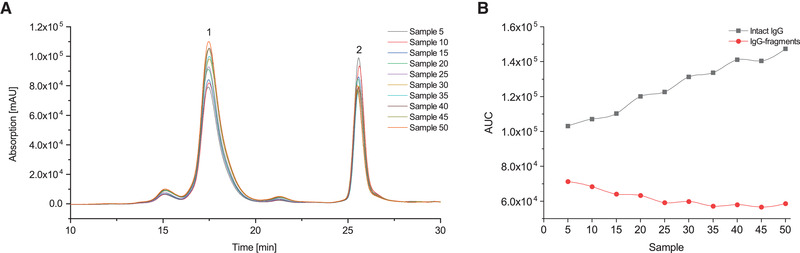
SEC‐HPLC spectra of the IgG‐hydrolysate samples 5–50 with Peak 1: Intact IgG antibody and Peak 2: IgG‐fragments (A) and AUC of intact IgG antibody peak and IgG‐fragments peak of the samples 5–50 (B). Sample size is 440 μL at a collection time of 40 min per sample. A total volume of 22 mL is collected over 33.5 h

The continuous enzymatic cleavage of IgG could be observed in a SEC‐HPLC analysis over a total time of 33.5 h. A total volume of 22 mL was collected in 50 individual and subsequent samples. For comparison of the yield over time the AUC of the intact IgG and the IgG‐fragments peak in the chromatogram was determined via integration as this equates the concentration of intact IgG and IgG‐fragments in the sample. For quantification of the overall yield the AUC of the intact IgG and the IgG‐fragments peak of sample 5 was compared to the AUC of the respective peaks in an IgG standard (1 mg mL^−1^). It could be observed that the AUC of the intact IgG peak increases over time. In line with this observation, the AUC of the cleaved IgG‐fragments peak decreases by 8.24 % over a period of 33.3 h. This implies 8.24 % less IgG antibody is cleaved by the immobilized papain in the ceramic capillary membrane reactor over a period of 33.3 h. Nevertheless, a comparatively stable hydrolysis could be maintained for the duration of the entire process (Figure 4).

Even if the immobilization of papain improves the thermal stability in some studies, papain is still susceptible to thermal degradation, autoproteolysis and spontaneous unfolding even though immobilization minimizes this effect [27, 28]. It is reported that adding up to 200 mM cysteine during the hydrolysis process can further enhance both stability and activity of immobilized papain [29]. The activity of papain presupposes the formation of a thiolate‐imidazolium ion pair of Cys‐25 and His‐159 located in the active site of the enzyme [30]. As indicated in kinetic studies, thiols may activate papain by increasing the decomposition rate of the enzyme‐substrate complex but the activation mechanism of papain remains not yet fully clarified [31]. In a preliminary examination, four different cysteine concentrations were tested in the continuous reactor system to find the ideal concentration for this application. For each of the cysteine concentrations 0, 5, 10, and 20 mM a total of 2.2 mL of IgG‐solution were collected in 5 samples with a sample size of 440 μL each. The IgG‐fragment yield of sample 5 was quantified for each examined cysteine concentration. In this application the addition of 10 mM cysteine to the IgG‐solution led to the best yield of antibody cleavage fragments (40.45%) without having a negative impact on the SEC‐HPLC analysis. The addition of 5 mM cysteine led to a yield of 24.96%, adding no cysteine left most of the papain in an inactive form resulting in a yield of only 1.19%. Increasing the amount of cysteine to 20 mM did not lead to a significant higher yield of antibody cleavage products (45.18%) but did show double peaks and peak tailing in the SEC‐HPLC analysis. Based on these results cysteine addition above a concentration of 20 mM was not tested. A batch process performed under comparable conditions yielded 60.58% antibody cleavage products when the intact IgG was added to the papain solution after 160 min for 40 min which is a higher yield than 40.45% antibody cleavage products in sample 5 of the continuous process. Compared to fraction 50 of the continuous process which yielded 32.82% antibody cleavage products the batch process was not able to convert any intact IgG added to the papain solution after 32.9 h for 40 min. The immobilized papain had an activity loss of only 7.63% over a duration of 33.5 h whereas the papain in solution used in the batch process had lost 100% of the original activity in this time.

The analysis in the SEC‐HPLC was not capable of separating Fab and Fc‐antibody fragments due to their near‐identical size. It is therefore likely that Fc‐antibody fragments appear in the IgG‐Fab‐peak. The SEC method is however reported of being capable to separate IgG‐Fab and Fc‐antibody fragments with a difference in retention time of 1 min. This separation could not be observed in this study even though the analysis was carried out exactly as described in literature. A wider peak size compared to the application manual could be observed in the SEC‐HPLC analysis which may have resulted in an overlap of the Fab‐ and Fc‐fragments peak [26].

All in all, the performed experiments prove the principal suitability of the CCCMRS for continuous fragmentation processes.

### 3.2 Continuous hydrolysis of pea and almond protein isolates

**FIGURE 5:**
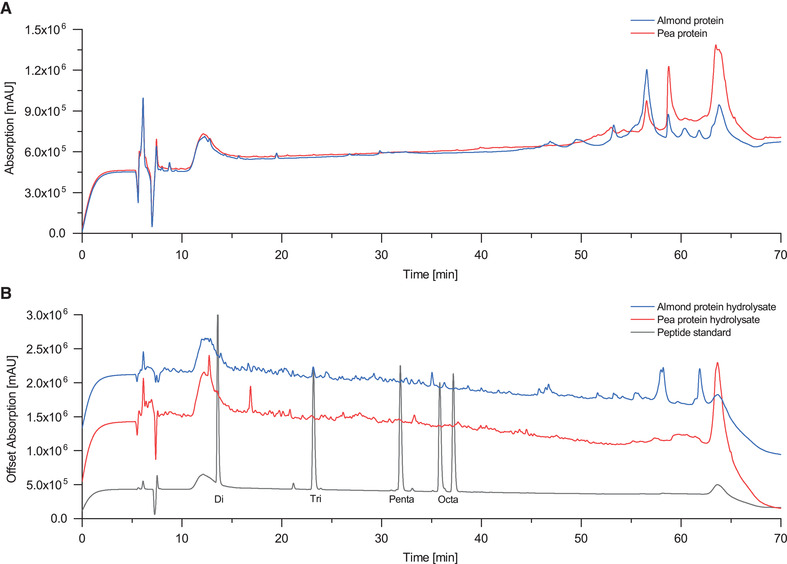
RP‐HPLC spectra of almond protein isolate and pea protein isolate (A) and almond protein hydrolysate, pea protein hydrolysate and peptide standard for size assignment (B)

**FIGURE 6:**
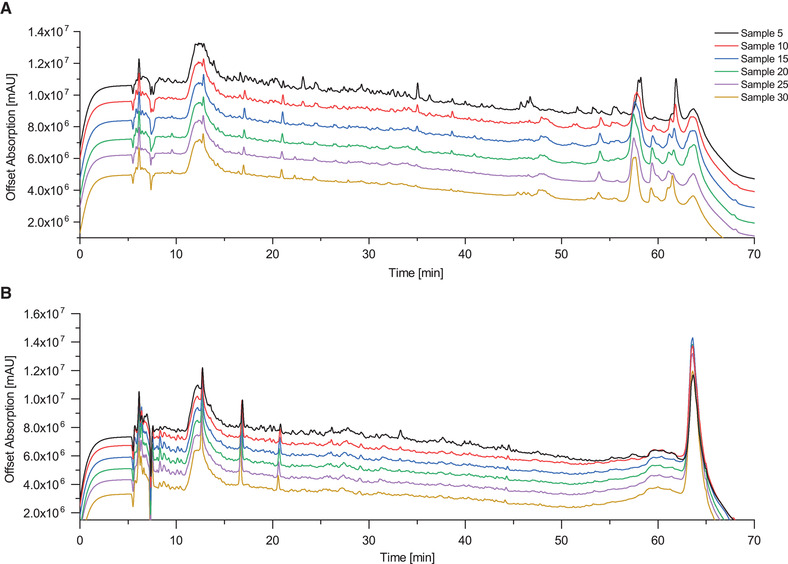
RP‐HPLC spectra of almond hydrolysate (A) and pea hydrolysate samples 5–30 (B). Sample size is 990 μL at a collection time of 90 min per sample. A total volume of 29.7 mL is collected over 45 h

In the second application the CCCMRS was employed to continuously generate protein hydrolysates from conventional food proteins. The analyses of Alcalase‐digested pea and almond‐protein hydrolysates in the RP‐HPLC show a homogenous peptide fingerprint with numerous small peaks comprising peptides ranging from a chain length of 2 up to a size of approximately 30 amino acids. Pea protein isolate contains the globulin storage protein fractions consisting of legumin, vicilin and convicilin; they make up 70–80% of the total protein content. The remaining 20–30% belong to the group of albumins which are metabolic proteins [32]. Almond major protein (AMP), also referred to as amandin, represents 65–70% of the total protein content of almond protein isolate. It is a storage protein belonging to the group of 14S globulins. Almond albumin fraction constitutes 20% of the almond protein isolate while at only 10%, the glutenin protein fraction contributes the smallest amount to the total protein content of almond protein isolate [33]. Considering the protein composition of pea‐ and almond protein isolate in the RP‐HPLC spectra (Figure 5), the large peaks appearing after 50 min can be assigned to the native proteins. They can also be partially found in the corresponding hydrolysates due to the incomplete digestion of the native proteins contained in pea and almond isolate. For quantification of the overall yield the AUC of all peaks appearing after 50 min of sample 5 was compared to the respective AUC of these peaks in the pea‐ and almond protein isolate solution. A total of 76.28% of the native proteins in the pea protein isolate were converted to peptides of different lengths in the pea protein hydrolysate. The conversion was 82.58% for the native proteins in the almond protein isolate. Batch processes performed under comparable conditions converted 83.57% of the native pea proteins and 89.52% of the native almond proteins into peptides of different lengths when adding pea‐ and respectively almond protein isolate to the Alcalase solution after 6 h which is a slightly higher conversion rate than in sample 5 of the continuous process. Comparing sample 30 of the continuous process to a batch process where pea‐ and respectively almond protein isolate were added to the Alcalase solution after 43.5 h for 90 min the batch process only converted 38.27% of the native pea protein and 42.42% of the native almond protein. In the continuous process 52.61% of native pea protein and 60.08% of native almond protein were converted into peptides of different lengths in sample 30 which was collected after 45 h. The immobilized Alcalase had an average activity loss of 23.08% over a duration of 45 h for pea‐ and almond protein isolate hydrolysis whereas the Alcalase in solution used in the batch process lost an average activity of 46.2% over this amount of time. Regarding the long‐term stability of the hydrolysis, it can be seen that the peptide fingerprint of the different samples is highly consistent. Nevertheless, more distinct peaks could be observed for the first five samples. This effect is more obvious in the almond protein hydrolysate samples where not only a higher rate of protein aggregation in the heated ceramic capillary membrane reactor was observed, but also on the capillary membrane surface. Aggregation can be a critical aspect when working with food protein isolates. Aggregated proteins have a decreased digestibility and tend to form a film on the surface of the ceramic capillary membranes resulting in a negative impact on the digestion process [34]. The temperature and the duration of the digestion process have the largest impact on protein aggregation. Mainly, protein aggregation could be observed in the ceramic capillary membrane reactor which is heated to 40°C to ensure sufficient protease activity. Keeping the temperature low wherever possible and additionally stirring the protein feed solution was able to prevent protein aggregation outside of the ceramic capillary membrane reactor. Therefore, the continuous hydrolysis process of food proteins can always be considered a tradeoff between protein aggregation and protease activity [35]. The rate of protein aggregation clearly depends on the hydrolyzed food protein and seems to occur mostly in the early phase of the hydrolysis process. Nevertheless, a stable hydrolysis process could be achieved over a notable timespan of 45 h producing 29.7 mL of hydrolysate in both tested digestion processes. The almond protein isolate seems to be more prone to aggregation than that of the pea protein isolate. The stability of immobilized Alcalase over time can be considered excellent and could also be verified in this study [36] (Figure 6).

## 4 CONCLUDING REMARKS

The CCCMRS has proven general applicability for continuous hydrolysis processes which are enzyme‐catalyzed and was able to evade typical difficulties that occur when working with proteases in solution. Experiments were able to show that the CCCMRS is capable of generating IgG‐antibody fragments from intact IgG antibody as well as a homogenous spectrum of peptides from two different food protein isolates with a chain length range from two up to approximately 30 amino acids and in addition showing considerable long‐term stability. Studies on the enzyme activity using chromogenic substrates did show reduced activity of immobilized proteases which could also be observed in a similar extent in the hydrolysis of pea‐ and almond protein as well as in the IgG‐cleavage process. On the other side autoproteolysis of proteases in solution did lead to a high rate of inactivation over time. This matches the observations stated in the literature [21, 22]. Nevertheless, the reduced activity of immobilized proteases is outweighed by the long‐term performance in the presented applications. The HPLC methods used for analyzing the cleavage samples are reported to be both reliable and highly sensitive methods for peptide and antibody analytics [37]. The limit of SEC‐HPLC in the detection of antibody fragments was clearly visible in the separation of the Fab‐ and Fc‐fragments due to their similar size even though the method is reported effective for the separation of antibody Fab‐ and Fc‐fragments in the literature [26]. In order to further improve the analysis of IgG cleavage, the SEC‐HPLC method has to be further optimized concerning the separation performance of Fab‐ and Fc‐fragments. Adding cysteine to the entire hydrolysis process did significantly enhance the stability and activity of the immobilized papain as reported in the literature [29]. Preliminary examinations showed that exceeding concentrations of 10 mM of cysteine did not further improve activity and stability significantly but did lead to difficulties in the SEC‐HPLC analysis. A concentration of 10 mM cysteine was therefore considered the ideal amount.

The ability to produce larger quantities of different food protein hydrolysates with a stable peptide fingerprint means that further studies can now include fractionation of peptides using preparative FPLC. The collected and purified peptide fractions can subsequently be tested in bioactivity assays. There have been numerous studies focusing on the production of BAPs from food protein origins. Since a great number of food proteins and proteolytic enzymes are suitable for the generation of BAPs, this field of scientific research is inexhaustible [38].

The CCCMRS was successfully utilized for both model processes. The long‐term stability of the investigated processes showed promising results as an efficient continuous hydrolysis could be carried out for over 24 h each.

Next steps in future studies will include bioactivity tests using the already produced protein hydrolysates after fractionation and purification as well as generating new potentially bioactive peptides from other food protein sources. Additionally, the versatile CCCMRS will be utilized to examine other suitable applications including the maturation of precursor proteins (e.g. Fibrin/Fibrinogen) and the removal of protein fusion domains such as His‐ or GST‐protein‐tags.

## CONFLICT OF INTEREST

The authors have declared no conflict of interest.

No experiments involving animals or humans were performed in the context of this work.

## NOMENCLATURE

[Ø] diameter

## Data Availability

The data that support the findings of this study are available from the corresponding author upon reasonable request.
